# Robot-Assisted Simple Hysterectomy Complicated by Postoperative Pelvic Hematoma Uncovering Platelet Dysfunction in Myelodysplastic Syndrome

**DOI:** 10.7759/cureus.91726

**Published:** 2025-09-06

**Authors:** Takeshi Motohara

**Affiliations:** 1 Department of Obstetrics and Gynecology, Faculty of Life Sciences, Kumamoto University, Kumamoto, JPN

**Keywords:** high-grade squamous intraepithelial lesion, myelodysplastic syndrome (mds), pelvic hematoma, platelet dysfunction, robot‑assisted simple hysterectomy

## Abstract

Postoperative hemorrhage following robot-assisted simple hysterectomy (RASH) is uncommon but can be life-threatening. While most cases are attributable to surgical factors, occult hematologic disorders may cause bleeding that is refractory to standard intraoperative hemostasis. A 48-year-old woman with a longstanding history of systemic lupus erythematosus (SLE) underwent RASH for a high-grade squamous intraepithelial lesion (HSIL/CIN3). Although mild intraoperative oozing was observed, there was no major vascular injury. Postoperatively, she developed a large pelvic hematoma with progressive hemoglobin decline, which ultimately led to delayed-onset contrast extravasation requiring transcatheter arterial embolization (TAE). Hematologic evaluation revealed giant platelets and impaired platelet aggregation, and bone marrow biopsy demonstrated megakaryocytic dysplasia, establishing a diagnosis of myelodysplastic syndrome (MDS). She had no preoperative cytopenias or bleeding history, and conventional coagulation testing failed to identify her hemostatic vulnerability. This case highlights the insidious nature of qualitative platelet dysfunction in MDS, which can precipitate significant perioperative hemorrhage even in the absence of thrombocytopenia. Multidisciplinary management involving gynecologic surgery, hematology, and interventional radiology was essential to achieve hemostasis and a favorable outcome. Clinicians should maintain a high index of suspicion for occult bleeding diatheses when confronted with unexplained perioperative hemorrhage following minimally invasive procedures.

## Introduction

Robot-assisted surgery has become increasingly prevalent in the field of gynecology, offering enhanced surgical precision, reduced blood loss, and faster recovery compared to conventional open surgery [1]. In the field of gynecology in Japan, robot-assisted simple hysterectomy (RASH) has been eligible for public insurance coverage since April 2018 for benign gynecologic conditions and early-stage endometrial cancer [2]. This approval has supported the steady expansion of minimally invasive robotic procedures in clinical gynecologic practice, making RASH a widely adopted approach for appropriately selected patients.

Despite its advantages, postoperative hemorrhage remains a clinically significant, albeit rare, complication of minimally invasive gynecologic surgery [3]. While most cases are attributable to surgical technique or anatomic challenges, underlying coagulopathies, including occult hematologic disorders, should be considered, particularly in cases of unexplained or refractory perioperative bleeding [4].

Myelodysplastic syndrome (MDS) is a clonal hematopoietic stem cell disorder characterized by ineffective hematopoiesis and a variable clinical course [5]. Although cytopenias are a hallmark of MDS, bleeding tendency may still occur due to qualitative platelet dysfunction, even when platelet counts are preserved and without overt systemic symptoms, including petechiae or bruising [6]. Such qualitative platelet dysfunction is frequently overlooked perioperatively and may result in serious hemorrhagic complications, particularly in relatively younger patients, where suspicion for hematologic disorders is typically lower.

Herein, we report a rare case of a postoperative pelvic hematoma following RASH for high-grade cervical intraepithelial neoplasia (HSIL/CIN3), in which subsequent evaluation revealed previously undiagnosed MDS with platelet dysfunction. This case highlights the importance of both hemostatic assessment and multidisciplinary management in the perioperative care of gynecologic robotic surgery.

## Case presentation

A 48-year-old nulligravida woman with a 12-year history of systemic lupus erythematosus (SLE) was referred to our institution for surgical management of HSIL/CIN3. She had been on a stable regimen of oral prednisolone (2 mg/day) and tacrolimus (2 mg/day) for SLE.

**Figure 1:**
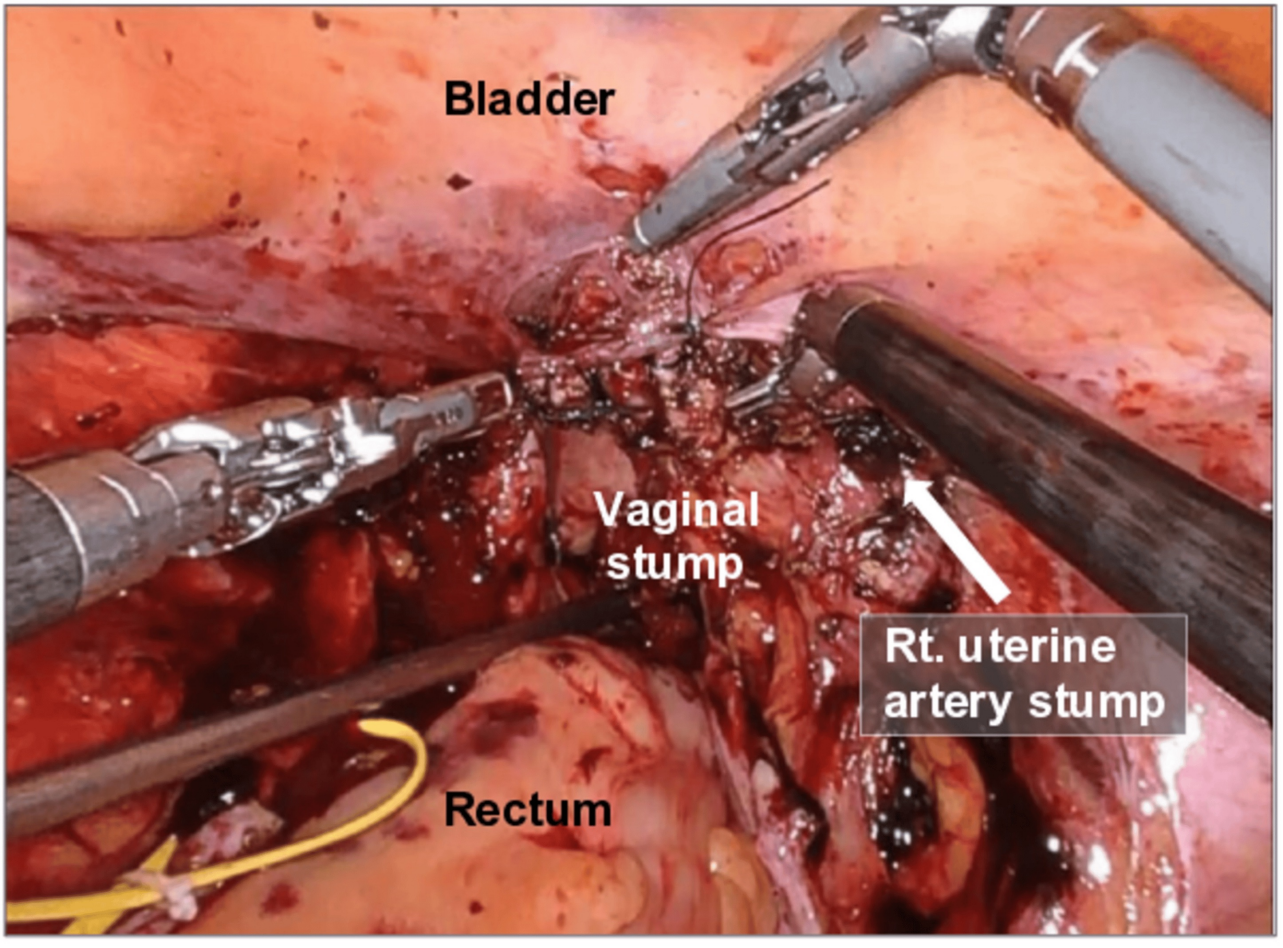
Intraoperative view during robot-assisted simple hysterectomy (RASH). Refractory oozing from the retroperitoneal space was observed despite careful dissection and hemostasis, with no identifiable major vascular injury. The uterine artery was meticulously transected using a vessel sealing device, and the stump of the right uterine artery is indicated (arrow).

She underwent RASH with bilateral salpingectomy. During the procedure, particularly throughout the retroperitoneal space exploration, persistent oozing exceeding the typical surgical expectation was observed despite meticulous hemostasis and careful coagulation (Figure 1). No major vascular injury was identified. No major vascular injury was identified. The operation lasted four hours and 51 minutes, with an estimated intraoperative blood loss of 40 mL. Although the blood loss was within the expected range for RASH, the operative time was prolonged by approximately two to three hours compared to the typical duration, reflecting the persistent intraoperative oozing. On the immediate postoperative blood test, the patient’s hemoglobin and platelet levels declined from 13.2 (normal range: 11.6-14.8 g/dL) to 10.9 g/dL and from 102,000 (normal range: 158,000-348,000/μL) to 87,000/μL, respectively. 

**Figure 2:**
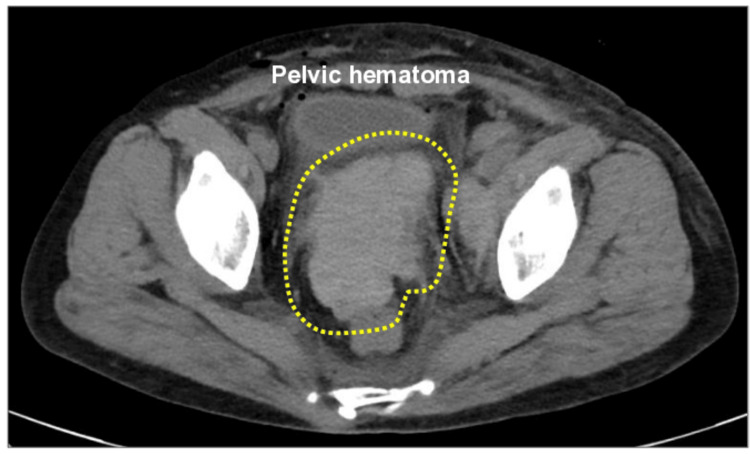
Postoperative pelvic hematoma without active bleeding. Contrast-enhanced computed tomography on postoperative day 3 showing a 7-cm pelvic hematoma (dotted outline) extending from the vaginal cuff toward the right uterine artery region, without evidence of contrast extravasation at this stage.

On postoperative day 3, the patient’s hemoglobin level had declined to 6.8 g/dL, accompanied by a decrease in platelet count to 59,000/μL, in the absence of overt vaginal bleeding or hemodynamic instability. At this point, no transfusion was given; instead, intravenous iron supplementation and careful observation were undertaken. Contrast-enhanced computed tomography (CT) at that time revealed a 7 cm pelvic hematoma extending from the vaginal cuff to the region surrounding the right uterine artery, with no evident contrast extravasation (Figure 2). 

On postoperative day 10, hemoglobin further declined to 5.1 g/dL, while the platelet count had paradoxically increased to 231,000/μL. This was considered a reactive phenomenon, possibly related to postoperative inflammatory response and iron metabolism. At this point, multiple transfusions of red blood cells (RBCs) and fresh frozen plasma (FFP) were administered, and CT-guided percutaneous drainage was performed, yielding a total of 350 mL of old blood. A drain was left in place for continuous drainage. Thereafter, the drain was removed on postoperative day 18, when the output had decreased to less than 10 mL per day.

**Figure 3:**
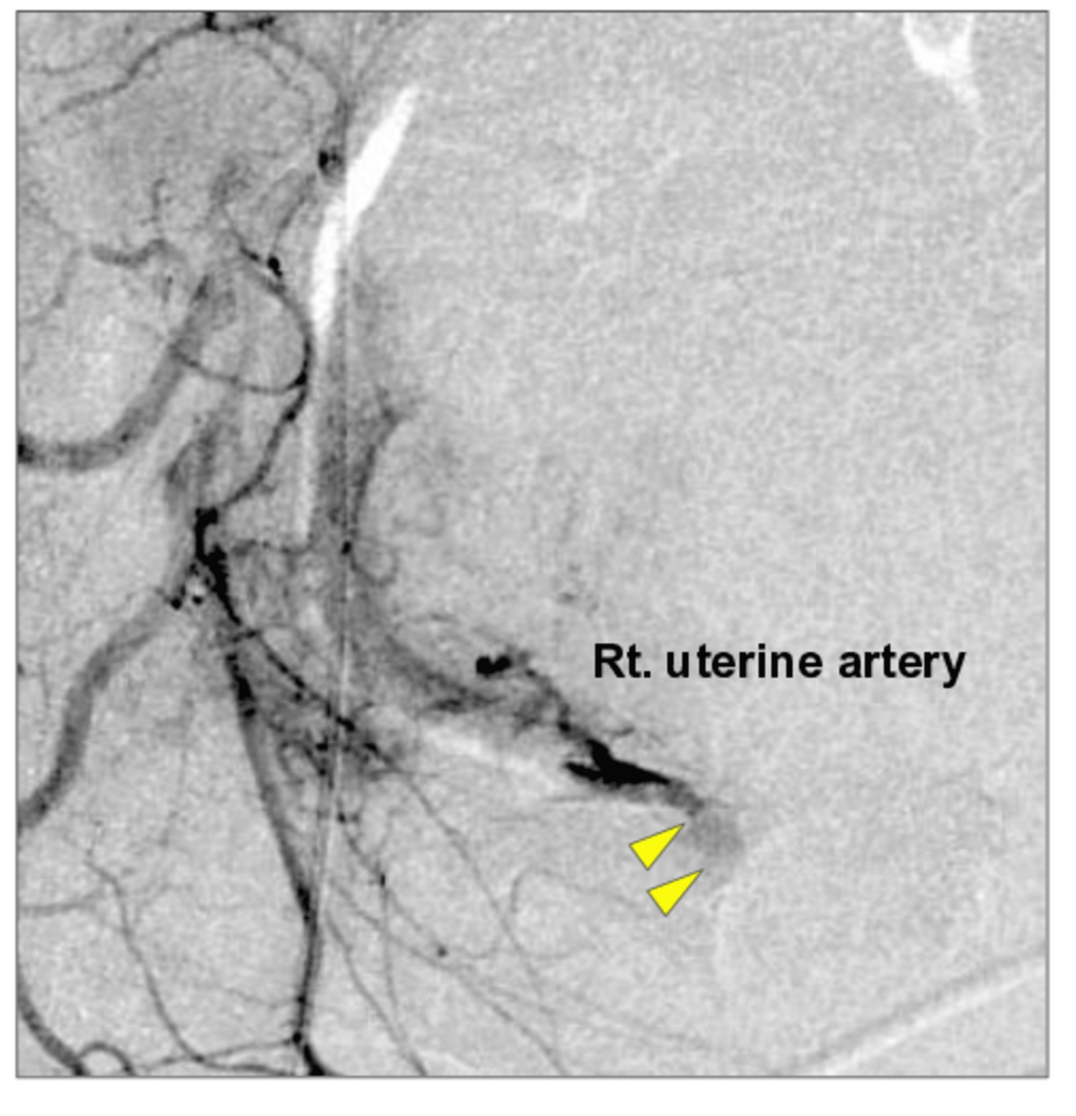
Delayed contrast extravasation requiring transcatheter arterial embolization (TAE). Follow-up contrast-enhanced computed tomography on postoperative day 18 demonstrating delayed-onset contrast extravasation from the right uterine artery (arrowhead). Emergency TAE was performed, achieving complete hemostasis.

Subsequently, on the same day, follow-up CT after drain removal revealed contrast extravasation near the right uterine artery for the first time. Emergency transcatheter arterial embolization (TAE) was successfully performed, resulting in complete hemostasis (Figure 3). 

In light of the unusually persistent intraoperative oozing observed during the procedure and the suboptimal hemostatic response noted in the postoperative course, the patient was referred to a hematologist. She subsequently underwent a comprehensive hematologic evaluation, including peripheral blood smear and platelet aggregation testing. These studies revealed giant platelets with impaired aggregation in response to ADP and collagen, confirming qualitative platelet dysfunction. Ultimately, bone marrow biopsy demonstrated an increased frequency of micromegakaryocytes and dysplastic megakaryocytes with separated round nuclei, establishing a definitive diagnosis of MDS. The diagnosis was established according to the 2022 WHO classification of myelodysplastic neoplasms [7]. Cytogenetic testing was performed and revealed no high-risk karyotypic abnormalities, while next-generation sequencing was not conducted at the time of diagnosis.

Based on these results, a management strategy of active surveillance was initiated, as the patient exhibited no high-risk features and had no postoperative symptoms. She remains under close hematologic follow-up, with no signs of clinical or cytogenetic progression to date.

## Discussion

This case underscores the clinical challenge of managing unexpected and refractory perioperative bleeding in minimally invasive gynecologic surgery. RASH is associated with a low incidence of significant hemorrhage. In fact, postoperative bleeding after minimally invasive hysterectomy has been reported in only 0.5-1.5% of cases in large series [3]. Nevertheless, occult systemic hematologic disorders such as MDS can amplify even standard surgical manipulation into substantial hemorrhagic events [6].

MDS typically occurs in the elderly but may also arise in younger individuals, particularly those with autoimmune conditions such as SLE, although this association is uncommon [8]. Previous studies have suggested that underlying immune dysregulation may contribute to the co-occurrence of MDS and SLE [9]. In the present case, the patient’s background treatment for SLE with prednisolone and tacrolimus was reviewed. Since tacrolimus has been associated with immune thrombocytopenia (ITP), ITP was considered in the differential diagnosis but was excluded based on the hematologic evaluation. In addition, the potential influence of her immunosuppressive regimen on hemostasis was considered; however, based on the clinical course, drug-related adverse effects were deemed unlikely in this case.

MDS has been recognized as a condition that can impair hemostasis due to ineffective hematopoiesis, even when routine blood counts appear acceptable [6]. In fact, the absence of overt thrombocytopenia may have masked underlying platelet functional abnormalities in this patient. Notably, platelet dysfunction in MDS often results from qualitative defects in adhesion, aggregation, or secretion due to megakaryocyte dysplasia, rather than platelet quantity alone [5,6]. As a result, standard preoperative coagulation screening often fails to identify these subtle yet clinically significant defects. In the present case, a baseline peripheral blood smear was not performed preoperatively, as the patient exhibited no cytopenias or prior bleeding history, which may have contributed to the delayed recognition of the underlying disorder.

From a clinical management standpoint, this case highlights the need to suspect platelet dysfunction in the setting of unexpected surgical oozing, even when routine screening for MDS is not warranted in asymptomatic individuals [10,11]. A multidisciplinary approach involving gynecologic surgeons, hematologists, and interventional radiologists was essential to achieve hemostasis and secure a favorable outcome.

While robotic-assisted surgery offers excellent precision and minimal invasiveness [12, 13], its commonly used vessel sealing systems without adjunctive suture ligation may occasionally be insufficient in achieving complete hemostasis in patients with underlying bleeding diathesis. In such cases, open surgical approaches-providing tactile feedback and allowing for conventional suture ligation-can serve as a complementary option for achieving more reliable vascular control. Recognizing the strengths and limitations of each approach is essential when selecting surgical strategies and evaluating perioperative bleeding risks.　

In this case, the sequence of clinical events - a progressive decline in hemoglobin level, formation of a sizable pelvic hematoma, and delayed-onset contrast extravasation requiring embolization - mirrored a concealed bleeding diathesis rather than simple postoperative ooze. The hematologic evaluation revealed platelet dysfunction and megakaryocytic dysplasia, eventually leading to the diagnosis of MDS. Notably, the patient had no prior history of cytopenia or bleeding episodes in the preoperative period, further underscoring the insidious nature of the disease.

To our knowledge, this is the first reported case of MDS-related bleeding diathesis revealed following RASH. This case also emphasizes the need for tailored preoperative hematologic evaluation in patients with known autoimmune conditions or subtle hemostatic abnormalities. MDS-related hemorrhagic events, although typically rare in the perioperative setting, should nevertheless be considered in the differential diagnosis when routine surgical explanations are insufficient. In similar patients with relatively young-onset MDS and concomitant autoimmune disease such as SLE, particular vigilance is warranted when performing robot-assisted gynecologic surgery.

## Conclusions

This case highlights the importance of considering potential bleeding diathesis, including MDS-related platelet dysfunction in the differential diagnosis of perioperative hemorrhage, particularly when bleeding is disproportionate or refractory to standard interventions. Early recognition and multidisciplinary management are critical to optimizing outcomes and preventing long-term complications. Enhanced perioperative screening in selected patients and collaboration across specialties remain key to safe surgical outcomes in complex comorbid cases.
